# Untargeted metabolomic profiling for identifying systemic signatures of *helicobacter pylori* infection in a guinea pig model

**DOI:** 10.1038/s41598-025-98016-w

**Published:** 2025-04-15

**Authors:** Weronika Gonciarz, Lucyna Kozlowska, Joanna Róg, Magdalena Chmiela

**Affiliations:** 1 Department of Immunology and Infectious Biology, Faculty of Biology and Environmental Protection, University of Lodz, 12/16 Banacha St., 90‑237 Lodz, Poland; 2 Laboratory of Human Metabolism Research, Department of Dietetics, Institute of Human Nutrition Sciences, Warsaw University of Life Sciences, 02-776 Warsaw, Poland

**Keywords:** Immunology, Infectious diseases, Helicobacter pylori

## Abstract

Infections caused by the Gram-negative bacterium *Helicobacter pylori* (*H. pylori*) can lead to gastritis, gastric or duodenal ulcers, and even gastric cancer in humans. Investigating quantitative changes in soluble biomarkers associated with *H. pylori* infection offers a promising method for monitoring the progression of the infection, inflammatory response and potentially systemic consequences. This study aimed to identify, using an experimental model of *H. pylori* infection in guinea pigs, the specific metabolomic biomarkers in the serum of *H. pylori*-infected (32) versus uninfected (32) animals. The *H. pylori* status was confirmed through histological, molecular, and serological examinations. Metabolomic profiling was conducted using UPLC-QTOF/MS methods. The metabolomic biomarkers significantly associated with *H. pylori* infection were selected based on volcano plots and traditional univariate receiver operating characteristics (ROC). This study identified 12 unique metabolites significantly differentiating *H. pylori*-infected guinea pigs from uninfected ones. In summary, the metabolomic profiling of serum samples, in combination with ROC characteristics of the data, enhances the monitoring of *H. pylori* infection and related inflammatory responses in guinea pigs experimentally infected with these bacteria, with potential applications in humans for prediction the infection course and its systemic effects.

## Introduction

Untargeted metabolomics is a widely used experimental biological tool that allows the assessment of the metabolic responses of an individual organism or population to drug treatment, an understanding of the relationship between the host factors and virulence factors of infectious agents as well as disease markers and facilitates the improvement of medical care for the patient^1^**.** The individual metabolic profile is determined by both host genetic factors and environmental factors^2^**.** Metabolomic research has the potential to aid in the discovery of selected biomarkers in different biological samples, e.g., blood, plasma, serum, urine, stool, and tissues during common and rare diseases, and improving diagnostics as well as treatment monitoring^1^**.** For instance, in biomedicine, analysis of metabolites using nuclear magnetic resonance (NMR), gas chromatography-mass spectrometry (GC–MS), flow injection analysis-mass spectrometry (FIA-MS), liquid chromatography-mass spectrometry (LC/MS) methods have been successfully applied to analyze the profile of obese patients^3^**,** diabetes patients^4^**,** as well as patients with cardiovascular disease^5^**,** or cancer^6^**.** Various metabolites are strictly linked to multiple cellular processes, and disturbances in cell physiology due to infectious or noninfectious diseases, which often result in altered metabolic footprints^7^**.**

Infection caused by the Gram-negative bacterium *H. pylori* affects approximately 50% of the world’s population, and in about 80% of infected people infection is asymptomatic^8,9^**.** Approximately 20% of infected people will develop clinical symptoms such as gastritis, duodenitis or peptic ulcer disease, and 1% of infected people will develop gastric cancer. In addition, *H. pylori* infection may influence the development of systemic diseases indirectly due to the induction of chronic inflammation^10–12^**.** Such a varied course of *H. pylori* infections and often their asymptomatic nature make early diagnosing of such infections and monitoring related systemic effects difficult. Therefore, animal models may be a good choice for studying the pathogenesis of *H. pylori* infections from the initial step and selecting appropriate systemic biomarkers^13–17^**.** The guinea pig model is particularly convenient due to the similarity of hormonal and immune responses and the thymus and bone marrow physiology of this animal species and humans^18–21^**.** The guinea pig requires the food source of vitamin C^22^ and develops delayed-type hypersensitivity reactions**.** Furthermore, the guinea pig’s major histocompatibility complex (MHC) molecules are similar to those of the human leukocyte antigen complex^23^**.** These animal model has several homologies to group 1 human cluster differentiation (CD) 1 proteins expressed in lymphoid and non-lymphoid tissues^21^, which serve as antigen-presenting molecules for non-peptide antigens to T-cells during infections. Moreover, human and guinea pig have a similar pattern of interferon-gamma expression and inducible nitric oxide synthase during infection^24,25^**.** The guinea pig has been chosen as a model for studying infections related to interleukin (IL)-8 secretion, a chemokine linking the CXCR1 tissue receptor^26^**.** Also, IL-12 is similar in both species^27^**.** There is also a similarity between the sequence of the CD8 co-receptors of cytotoxic T lymphocytes. Moreover, guinea pigs and humans produce the Regulated Activation Normal T Cell Expressed and Secreted (RANTES) chemokine^28^**.** An essential advantage of using the guinea pig model for studying the course of *H. pylori* infection is that these animals are not naturally infected with different *Helicobacter* species^29^**.**

Previously we developed a model of experimental *H. pylori* infection in guinea pigs along with microbiological, serological, and molecular determinants of this infection^12,14–17^**.** Our present research has been focusing on finding systemic signatures of *H. pylori* infection in a guinea pig model by using ultra-performance liquid chromatography and quadrupole time-of-flight mass spectrometry (UPLC-QTOF/MS), which is a valuable platform for untargeted metabolomic analysis, giving the possibility of analysis for nonvolatile compounds and large or thermally unstable compounds. Untargeted metabolomics facilitates analysis of all detectable metabolites without relying on pre-specified categories, and then the selection of biomarkers for monitoring the course of *H. pylori* infection in guinea pigs. We expect that results obtained using this animal model will help to identify systemic biomarkers corelating with *H. pylori* infection in humans and predict its local and systemic consequences.

## Materials and methods

### Animal study

#### Ethics statement for animal study

All experiments involving animals were developed according to the Animal Research recommendations: Reporting of In Vivo Experiments (ARRIVE) guidelines and guidelines and regulations European Union (EU) directive (Directive 2010/63/EU of the European Parliament and of the Council of 22 September 2010 on the protection of animals used for scientific purposes (Dz.U. L 276 z 20.10.2010, s. 33–79) and were approved by the Local Ethics Committee (LKE9) for Animal Experiments of the Medical University of Lodz, Poland, which was established by the Ministry of Science and Higher Education in Poland (Ethics Committee decision number: 58/ŁB45/2016.

#### *H. pylor*i infection in *Caviaporcellus* (guinea pig)

Three-month-old male Himalayan guinea pigs (400–600 g), free of pathogens, were housed in the Animal House at the Faculty of Biology and Environmental Protection, University of Lodz (Poland), kept in cages with free access to drinking water and fed with standard chow. The animals were inoculated *per os* with 1 mL of freshly prepared bacterial suspension of the reference *H. pylori* strain CCUG 17874 (Culture Collection University of Gothenburg, Sweden) (10^10^ CFU/mL), three times in two-day intervals as previously described^14,15^**.** Then, 28 days after the last *H. pylori* inoculation, the animals were euthanized with an overdose of sodium barbiturate (Morbital, Biowet, Puławy, Poland), according to a protocol approved by an ethics committee. The gastric tissue was collected for analyses, whereas blood samples were processed to obtain serum and then stored at -80 °C. *H. pylori* infection was confirmed by microscopic imaging of *Helicobacter-*like organisms (HLOs) and scoring tissue inflammation in thin layer preparations stained by routine histological procedures (Giemsa staining, hematoxylin & eosin), detection of *H. pylori* by staining tissue specimens with anti-*H. pylori* antibodies fluorescently labeled, and using a polymerase chain reaction (PCR) to detect sequences encoding *H. pylori* cytotoxin associated gene A (CagA) protein and UreC subunit of urease, as previously described^14,15^**.** The laboratory version of enzyme-linked immunosorbent assay (ELISA) for detection of anti-*H. pylori* IgM and IgG antibodies against the *H. pylori* antigenic complex—glycine extract (GE), which was obtained by extraction with glycine acid buffer of surface antigens from the reference *H. pylori* CCUG 17874 strain, producing CagA and VacA proteins, was performed as previously described^30^**.** In total, 64 animals were used in the study: 32 uninfected (control) and 32 infected with *H. pylori.*

### Metabolomic analysis

#### Materials

LC–MS grade solvents: acetonitrile (ACN), methanol (MeOH), (J.T. Baker, Avantor Performance Materials, Gliwice, Poland) and LC–MS-grade mobile phase modifiers: formic acid (FA) (Chem-LAB NV, Zedelgem, Belgium) were applied. Ultra-high purity water was prepared by the R5 UV Hydrolab system (Wislina, Poland). Internal standard (IS) mixture reagents were benzoyl-D5 98% and L-phenylalanine 3,3-D2 98% (Cambridge Isotope Laboratories, Inc., Tewksbury, MA, USA).

#### Metabolomic procedures

1. Sample collection and processing

The blood samples, which were collected from *Cavia porcellus* to dry tubes, were stored for 60 min at 37°C and overnight at 4°C for clothing, and then 500 μL aliquots of serum were pipetted into safe-lock Eppendorf tubes (Eppendorf®, Hamburg Germany), and frozen at − 80 °C until further analysis. After thawing at 4 °C, serum samples were centrifuged at 20 000 × g for 10 min at 4 °C to remove cellular debris. Next, 100 μL of samples were transferred and mixed with the 300 μL ice-cold acetonitrile solution and IS. The diluted serum samples were incubated for 20 min at − 20 °C and centrifuged (20,000 × g, 10 min, 4 °C). Next, 200 µL of supernatant was aliquoted into low-recovery-volume HPLC vials. Quality control (QC) samples were prepared by mixing equal aliquots of all thawed and centrifuged serum samples. The analytical batch included 10 samples for system equilibration, 9 QC samples, 64 *Cavia porcellus* serum samples (32 from *H. pylori*-infected and 32 from non-infected animals), and 2 blank samples.

2. Instrumental analysis—UPLC-QTOF/MS analysis

For the assessment of serum metabolomic profile, an untargeted metabolomic analysis with the analytical system consisting of Waters Acquity™ UPLC (Waters Corp., Milford, MA, USA) connected to a Synapt G2Si QTOF/MS spectrometer (Waters MS Technologies, Manchester, UK), equipped with an electrospray source (ESI) (Waters MS Technologies, Manchester, UK) was used. Metabolite separation was executed using ACQUITY UPLC BEH C18 precolumn (1.7 µm, VanGuard Precolumn 2.1 × 5 mm) connected with an ACQUITY UPLC BEH C18 (1.7 µm, 2.1 × 100 mm) chromatography column (Waters, Milford, MA, USA) for positive and negative ionization modes. The mobile phases were as follows: (A) 0.1% FA in water and (B) 0.1% FA in ACN. The injection volume was 4 µL, the temperature was maintained at 40°C, and the flow was 3 mL/min (positive mode) and 2.5 mL/min (negative mode). Tables S1 and S2 show the optimized gradient elution procedures.

The same conditions were applied to both ESI + and ESI − modes. All analyses were performed in MS centroid, high-resolution mode with a time scan of 0.3 s. The gas flows were 900 L/h for the desolvation gas, 100 L/h for cone gas, and with nebulizer 6.5 Bar. Temperatures were 350°C desolvation and 120°C for the source. The voltage of the capillary was 3.2 kV (positive mode) and 2.4 kV (negative mode). To ensure accuracy and reproducibility, the lock mass (leucine-enkephalin) was used with the following settings: scan time 0.5 s., interval 15 s., scans to average: 3, and mass window ± 0.5 Da.

3. Data analysis (metabolomic data preprocessing, normalization, and annotation)

**Fig. 1:**
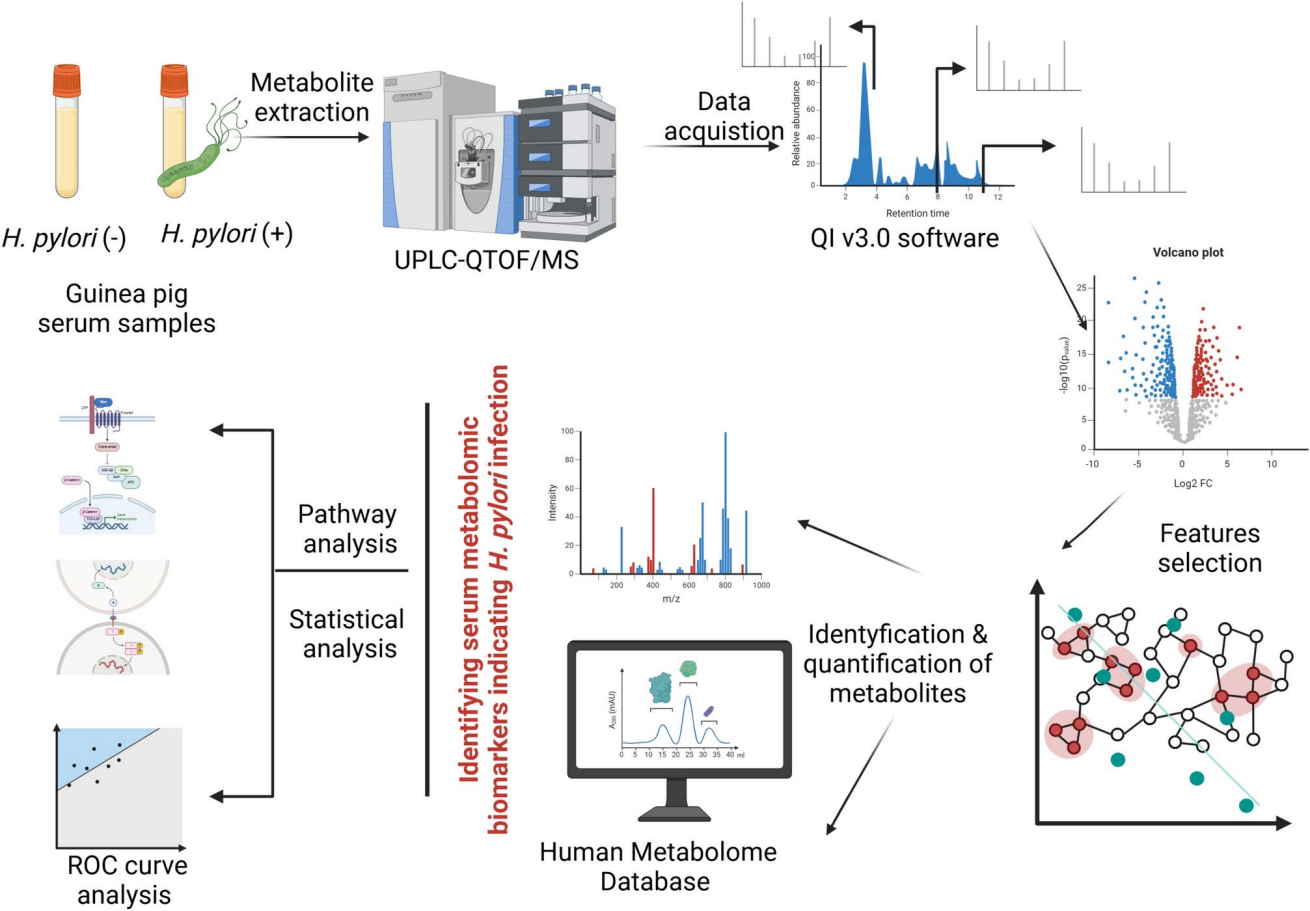
The steps of processing and analysis of the studied guinea pig serum samples using an untargeted metabolomic procedure. Created in BioRender. Mikolajczyk-Chmiela, M. (2025) https://BioRender.com/z87y026.

The principle of metabolite extraction, data acquisition, selection of features, identification and quantification of metabolites, and selection of metabolomic biomarkers differentiating *H. pylori* infected from *H. pylori* uninfected animals are shown in Fig. 1. The samples were initially tested to evaluate system stability. Following this, the noise signals were eliminated, interference from instability was tackled, and operational errors were corrected. Finally, the data were normalized and scaled for statistical analysis.

Progenesis QI v3.0 software (Waters, Milford, MA, USA) processed untargeted data files. We applied the default UPLC—High Resolution (Waters) parameter set to obtain the raw intensity data for further filtration. The metabolic features with a blank contribution greater than 10% and a relative standard deviation (RSD) of QC greater than 35% were removed. A Group Entropy-Based Web Platform, MetaboGroupS (https://www.omicsolution.com/wukong/MetaboGroupS/), was used to evaluate normalization methods of filtered raw data. Seven common normalization methods (median normalization, standard normalization, variance-stabilizing normalization, removal of unwanted variation-random normalization, QC sample-based support vector regression, EigenMS, and QC sample-based support vector regression) were evaluated. We chose the EigenMS method with minimal coefficients of variation (CV) of entropy in QC samples to normalize both positive and negative modes **(see Figures S1 and S2).** The Human Metabolome Database (HMDB) (https://www.hmdb.ca/) was used to compare resulting fragmentation spectra to annotate compounds.

### Statistical analysis

Statistica 13.3 PL software was used. The differences in the signal intensity of the putatively annotated metabolites between groups (*H. pylori-*infected group *vs*. *H. pylori* non-infected animals) were analyzed using the unpaired t-test (p-value threshold = 0.05). Fold Change (FC) analysis (FC threshold = 1.5), and these results were combined into one single graph as a volcano plot method with the MetaboAnalyst online platform (https://www.metaboanalyst.ca/). The results were compared to find potential metabolites that differentiated the examined groups. Classical univariate receiver operating characteristic (ROC) curve analyses were used to identify potential biomarkers for determining the analyzed groups. Classical ROC analysis is frequently applied to algorithms for building ROC curves and calculating the area under the curve (AUC) to compute optimal metabolite cutoffs and generate sensitivity and specificity data. These classical univariate ROC curve analyses were generated in the MetaboAnalyst 5.0 platform (https://www.metaboanalyst.ca) and accessed on 20 September 2024).

## Results

### ***H. pylori*** status of studied animals

**Fig. 2:**
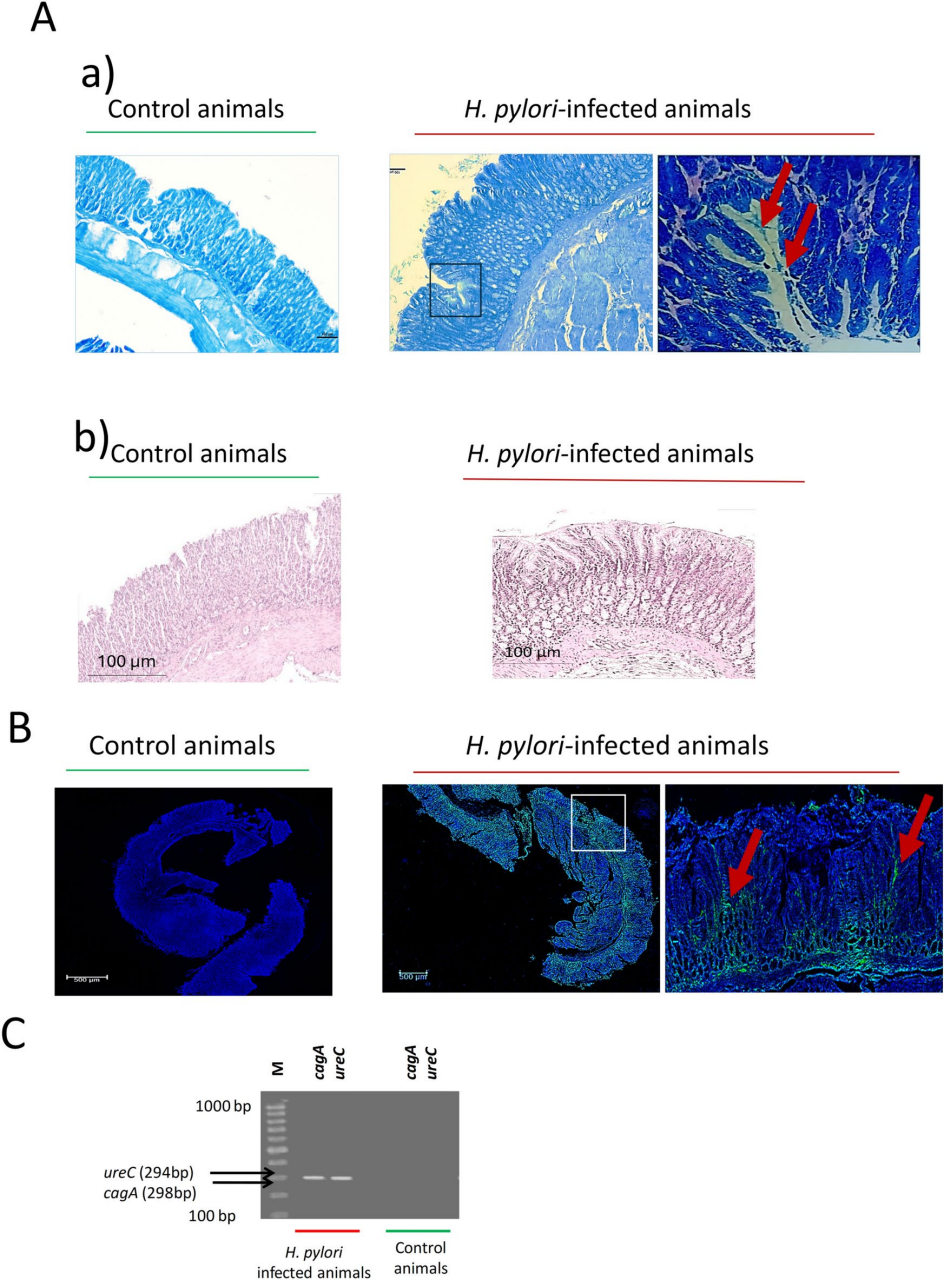
Histological and molecular confirmation of *H. pylori* infection in the studied guinea pig model.

In all animals (32/32) inoculated with *H. pylori*, the status of *H. pylori* infection was confirmed 28 days after the last inoculation by histological, molecular, and serological examination **(**Table 1**).** The gastric mucosa of guinea pigs inoculated with *H. pylori* was colonized by *Helicobacter*-like organisms (HLO), as shown by Giemsa staining (Fig. 2Aa). Tissue staining with hematoxylin and eosin showed infiltration of immune cells within the gastric mucosa (Fig. 2Ab). Staining of gastric tissue sections with anti-*H. pylori* antibodies fluorescently labeled confirmed the colonization with *H. pylori* of gastric tissue from animals inoculated with these bacteria but not control animals, which were not *H. pylori* inoculated (Fig. 2B).

**Table 1:** The *H. pylori* status of guinea pigs was confirmed by histological, molecular, and serological examination.

Sample	Diagnostic assay		Study groups (n = 64)	
			***H. pylori-negative***	***H. pylori-positive***
Gastric tissue	Histopathology- HLO	Giemsa staining	0/32*	32/32*
	PCR	*cagA*	0/32*	32/32*
		*ureC*	0/32*	32/32*
Serum	ELISA	IgG anti-*H. pylori*	0/32* (OD _λ450_ = 0.422** ± ** 0.043)*	32/32* (OD _λ450_ = 1.251 ± 0.050)*

HLO-*Helicobacter*-like organisms; *cagA*—cytotoxin associated gene A; *ureC* – the gene encoding urease subunit C. Shown are mean values of optical density (OD _λ450_ ) in enzyme-linked immunosorbent assay (ELISA) with standard deviation (SD). The differences between tested variables were assessed using Statistica 13.3 PL software with a nonparametric Mann–Whitney U test. The results were considered statistically significant when * p < 0.05: *H. pylori-*infected group vs. non-infected animals.

In the gastric tissue of *H. pylori* infected animals, *cagA and ureC* sequences encoding CagA protein and subunit C of urease, respectively, were detected by PCR (Fig. 2C). These sequences were not detected in 32 of 32 non-infected animals. The infected animals responded to *H. pylori* by producing anti-*H. pylori* immunoglobulins (Igs) of IgG class **(**Table 1**).**

A a– Representative microscopic images for Giemsa staining of gastric tissue of control and *H. pylori* inoculated guinea pigs; A b—Representative microscopic images for hematoxylin and eosin staining of gastric tissue of control and *H. pylori* inoculated guinea pigs from the light microscope (Nikon ECLIPSE 50i) using a Nikon Plan 10x / 0.25 Ph1 DR WD 10.5; or Nikon Plan 100XA / 1.25 Oil WD 0.2 objective. The marked box and arrows indicate the localization of *Helicobacter*—like organisms.

B –Representative microscopic images of gastric tissue of control and *H. pylori* inoculated guinea pigs stained with anti-*H. pylori* antibodies fluorescently labeled. The studied tissues were visualized in a confocal microscope (MICA WideFocal Live Cell Leica Microsystems, Frankfurt, Germany) using a 10x/0.32 or 40 × W/1.1 (water immersion) objective at the appropriate wavelengths for nuclear marker—DAPI (358 nm excitation, 461 nm emission) or fluorescein isothiocyanate—FITC (498 nm excitation, 517 nm emission). The Leica Application Suite X Office 1.4.7 28,921 software (https://www.leica-microsystems.com/products/microscope-software/p/leica-las-x-ls/downloads/), LAS X Office 1.4.7 28,921, Leica Microsystems,https://www.leica-microsystems.com/products/microscope-software/p/leica-las-x-ls/ Frankfurt, Germany) was used for cell imaging.

C- Representative products: *cagA* (298 bp) and *ureC* (294 bp) sequences amplified in polymerase chain reaction (PCR) loaded onto 1.4% agarose gel stained with ethidium bromide**.** M — 1000 base pair (bp) ladder—molecular weight marker.

### Untargeted metabolomic analysis of guinea pig serum samples – selection of potential biomarkers of *H. pylori* infection

**Fig. 3:**
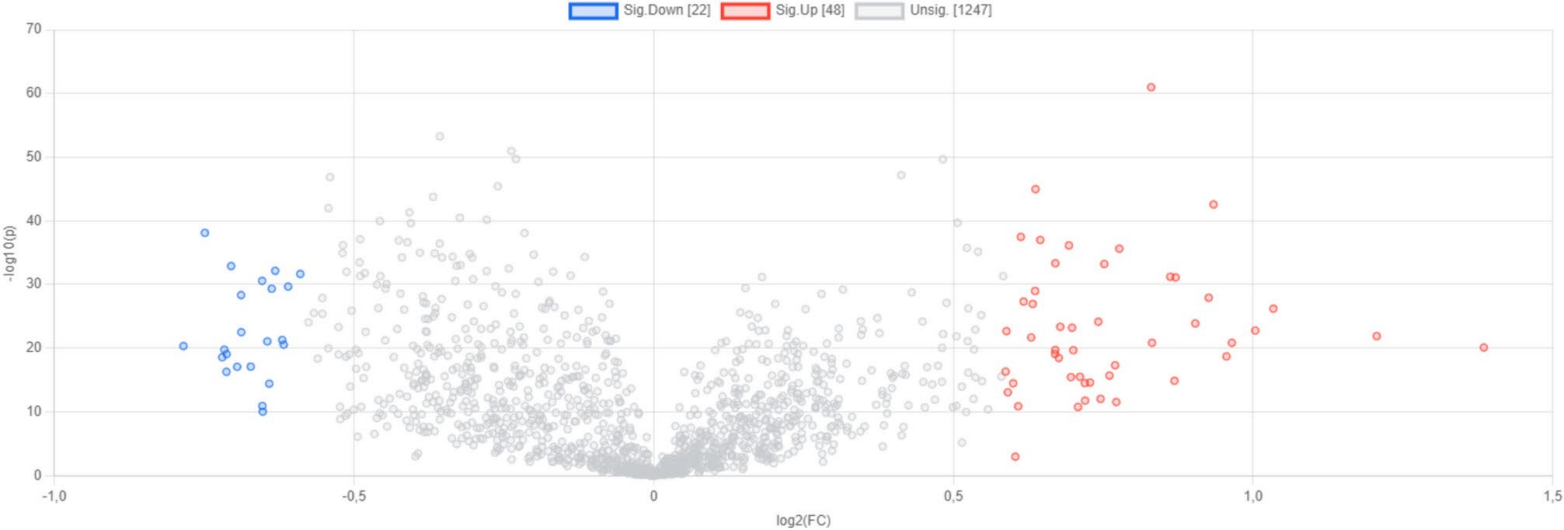
The volcano plot image shows changes in signal intensity corresponding to specific metabolites between *H. pylori*-infected *vs. H. pylori* uninfected animals. Metabolites with significantly higher signal: red; metabolites with significantly lower signal: blue.

The present study used the UPLC-QTOF/MS system to characterize serum samples based on systemic signatures of *H. pylori* infection in a guinea pig model. Fold change analysis and T-test results in *H. pylori*-infected *vs*. *H. pylori* uninfected animals facilitated the selection of 22 metabolites with a signal intensity significantly lower and 48 metabolites with a signal intensity significantly higher **(**Fig. 3**).** In Table 2, we selected 12 metabolites with significantly higher signals associated with *H. pylori* infection. Volcano plots **(**Fig. 3**)** and hierarchical clustering (Fi) of selected metabolites revealed significant differences between studied animal groups. MS/MS spectra of putatively annotated metabolites are presented in **Figure S3** in supplementary materials (our input spectra are shown on top in blue, and the closest matching result spectra are shown underneath in red).

**Table 2:** Putatively annotated metabolites, whose signal intensity was significantly increased in *H. pylori*-positive guinea pig serum samples *vs.* serum samples from *H. pylori*-negative animals.

Lp	log2(FC)	FC	*m*/*z*	RT (min)	*p*-Value	Name (ID in HMDB)	Cellular locations	Biological process
1	1.2063	2.31	368.2231	2.4	0.015061	20-dihydroxy leukotriene B4 HMDB0012635	Extracellular membrane	Lipid peroxidation Fatty acids metabolism Cell signaling Lipid metabolism pathway https://hmdb.ca/metabolites/
2	0.5568	1.94	394.2702	2.65	0.000024	PGD2 Dihomo-gamma-linolenoylethanolamide (DGLEA) HMDB0013629	Cytoplasm membrane	Lipid peroxidation Fatty acids metabolism Cell signaling Lipid metabolism pathway Inflammatory response https://hmdb.ca/metabolites/
3	0.7517	1.68	211.1444	2.2	0.001232	L,L-Cyclo(leucylprolyl) HMDB0034276	Extracellular cytoplasm	Tyrosine metabolism Alkaloid biosynthesis https://hmdb.ca/metabolites/
4	0.6172	1.50	765.5122	7.02	2.11E-23	(PC) Phosphatidylcholine HMDB0007999	Extracellular membrane	PC shows anti-inflammatory effects^31^.
5	0.7456	1.68	602.381	7.24	0.000000	Lysophospholipid (LPC) HMDB0010407	Extracellular membrane	LPC has several protective or anti-inflammatory effects^31^
6	0,6960	1.62	825.5384	7.43	3.45E-16	(PS) Phosphatidylserine HMDB0012402	Membrane extracellular	Metabolic pathway Phosphatidylserine biosynthesis Phospholipid biosynthesis Phosphatidylcholine biosynthesis Phosphatidylethanolamine biosynthesis https://hmdb.ca/metabolites/
7	0.6784	1.60	787.5268	6.98	4.41E-24	(PG) Phosphatidylglycerol HMDB0010656	Extracellular membrane	PGs have a net charge of -1 at physiological pH and are found in high concentrations in mitochondrial membranes and as components of pulmonary surfactant. PG also serves as a precursor for the synthesis of cardiolipin https://hmdb.ca/metabolites/
8	0.6758	1.60	204.1233	0.98	3.38E-19	N-Lactoylleucine HMDB0062176	Extracellular	Ubiquitous metabolites originate from CNDP2-mediated reverse proteolysis of lactate and amino acids^32^
9	0.6695	1.59	279.1705	2.68	7.72E-20	Iso leucyl-phenylalanine HMDB0028914	no data	Isoleucyl-phenylalanine is a dipeptide composed of isoleucine and phenylalanine. It is an incomplete breakdown product of protein digestion or protein catabolism. Some dipeptides are known to have physiological or cell signaling effects. However, most are short-lived intermediates on their way to specific amino acid degradation pathways following further proteolysis. https://hmdb.ca/metabolites/
10	0.6370	1.56	209.1286	2.66	1.05E-45	5-Methoxytryptophol HMDB0001896	Extracellular	5-Methoxytryptophol is an indoleamine synthesized from the pineal gland hormone serotonin and, together with melatonin, is involved with diurnal rhythms regulation^33,34^
11	0.6363	1.55	568.3494	2.9	1.03E-29	Deoxycholic acid 3-glucuronide HMDB0002596	Extracellular membrane	Membrane stabilizer Signaling molecule https://hmdb.ca/metabolites/
12	0.6081	1.52	86.0605	2.86	1.33E-11	Gamma-aminobutyric acid (GABA) HMDB0000112	Extracellular membrane	Metabolic pathway Glutamate metabolism 4-Aminobutanoate degradation Putrescine degradation II Arginine metabolism Ornithine metabolism Glutamic acid metabolism Butanoate metabolism https://hmdb.ca/metabolites/

Abbreviations: FC – fold change; m/z – mass to charge ratio; RT – retention time; HMDB – Human.

The metabolites well differentiating the guinea pigs infected with *H. pylori* from *H. pylori* uninfected animals included: 20-dihydroxy leukotriene B4 (HMDB0012635), PGD2 ethanolamide (HMDB0013629), L.L-Cyclo(leucylprolyl) (HMDB0034276), LysoPC(P-16:0/0:0) (HMDB0010407), PS(18:2(9Z.12Z)/18:2(9Z.12Z)) (HMDB0012402), PG(18:2(9Z.12Z)/22:4(7Z.10Z.13Z.16Z)) (HMDB0010656), N-Lactoylleucine (HMDB0062176), Isoleucyl-Phenylalanine (HMDB0028914), 5-Methoxytryptophol (HMDB0001896), Deoxycholic acid 3-glucuronide (HMDB0002596), PC(16:1(9Z)/14:1(9Z)), (HMDB0007999), gamma-Aminobutyric acid (HMDB0000112)** (**Table 2**).**

### Metabolome database

**Fig. 4:**
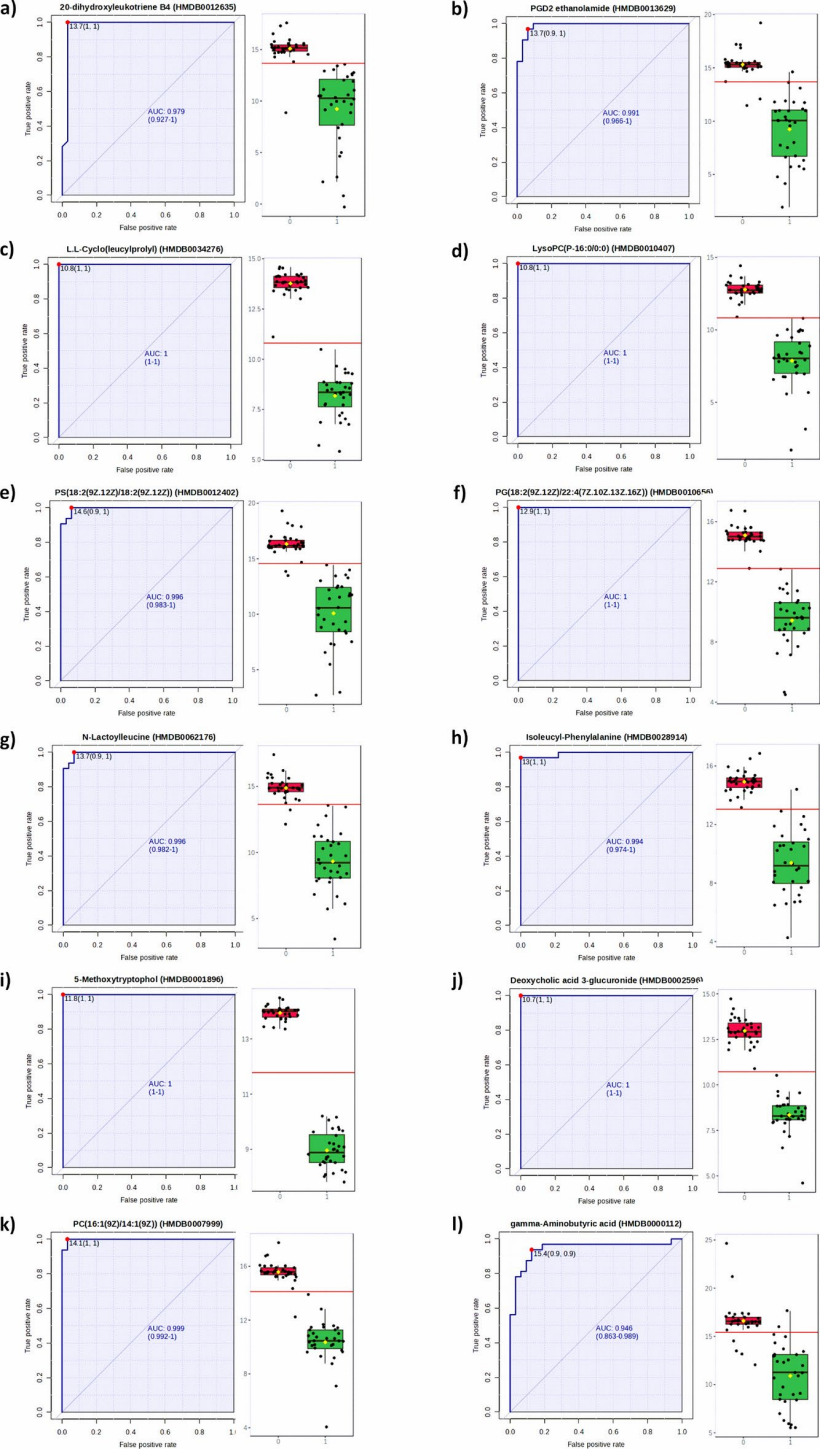
Classical univariate receiver operating characteristic curves generated from the spectral data to identify serum metabolomic biomarkers indicating *H. pylori* infection. Box plots representing the distribution of the normalized signal intensities come from: (**a**) 20-dihydroxyleukotriene B4 (HMDB0012635); (**b**) PGD2 ethanolamide (HMDB0013629); (**c**) L.L-Cyclo(leucylprolyl) (HMDB0034276); (**d**) LysoPC(P-16:0/0:0) (HMDB0010407); (**e**) PS(18:2(9Z.12Z)/18:2(9Z.12Z)) (HMDB0012402); (**f**) PG(18:2(9Z.12Z)/22:4(7Z.10Z.13Z.16Z)) (HMDB0010656); (**g**) N-Lactoylleucine (HMDB0062176); (**h**) Isoleucyl-Phenylalanine (HMDB0028914); (**i**) 5-Methoxytryptophol (HMDB0001896); (**j**) Deoxycholic acid 3-glucuronide (HMDB0002596);) (**k**) PC(16:1(9Z)/14:1(9Z)) (HMDB0007999); (**l**) gamma-Aminobutyric acid (HMDB0000112). The boxes represent the interquartile range (difference between the upper 75% and lower quartile 25%), the thick black lines, and the median. A horizontal line is in red, indicating the optimal cutoff. Abbreviations: 0—*H. pylori-negative*; 1 – *H. pylori* positive.

The classical univariate ROC curve analysis confirmed the usefulness of these selected biomarkers in the differentiation of *H. pylori* infected and *H. pylori* non-infected guinea pigs **(**Fig. 2**).** We observed that potential biomarkers of serum samples from *H. pylori* infected animals showed AUC higher than 0.9, which means the statistically significant difference between the studied groups (*H. pylori* infected *vs. H. pylori* non-infected guinea pigs) **(**Fig. 4**, Table S3).**

## Discussion

Diagnostic of *H. pylori* infections in humans is based on examination of gastric tissue specimens collected during gastroscopy. The gold standard of diagnostic tests contains a rapid urease test (RUT) for screening the activity of urease produced by *H. pylori*, histological staining of gastric tissue for detection of *Helicobacter*-like organisms (HLO), assessment of the inflammatory response, and microbiological culture of bacteria^35,36^**.** Non-invasive diagnostic methods include the ^13^C urea breath test (UBT), which is sufficiently specific and sensitive for primary diagnosis and for confirming the effectiveness of eradication therapy. Serological assays, such as enzyme-linked immunosorbent assay (ELISA), detect particular anti-*H. pylori* antibodies in serum samples or *H. pylori* antigens in stool samples^36,37^**.** However, a comprehensive method is needed to quantify selected markers to track the systemic effects of *H. pylori* local infection in gastric tissue. Potentially, such markers may help predict the course of infection and its regional and systemic consequences, including coronary heart disease, anemia, and type 2 diabetes^38–42^**.** Knowledge about the mechanisms driving different courses of *H. pylori* gastric infections and the potential role of these bacteria in developing systemic diseases is insufficient. Experimental animal models allow deepening this knowledge. We used the experimental model of *H. pylori* infection in guinea pig, which is accepted for studying the pathogenesis of *H. pylori* infection and comparing to some extent data derived from this model to *H. pylori* infection in human due to structural and functional similarity of the immune system and physiology of the gastroduodenal tract in both species.

Although serum antibody titer assay, urease activity assay, and stool antigen assay in conjunction with histological examination of gastric tissue may be sufficient for diagnosing *H. pylori* infection in humans, there are no reliable markers to differentiate the course of infection and its possible systemic consequences. Metabolomic analyses offer such opportunities. They have been applied to search for markers of infectious diseases and monitor treatment effectiveness. For instance, in plasma samples from subjects infected with human immunodeficiency virus (HIV), the LC–MS analysis showed the presence of acetate, citrate, creatine, dicarboxylicacylcarnitines, dopamine, glucose, glycerophospholipids, glycolysis, L-aspartate, plasmalogen/plasminogen, lysophospholipids, methylglutarylcarnitine, phosphatidylcholines, sphingomyelin, sphingosine-1-phosphate as potential biomarkers^43^**.** In serum samples from patients infected with influenza virus Banoei et al., using ^1^H-NMR, detected citrate; fumarate; 3-Methyl,2-Isovalerate; alanine; tyrosine; methionine; histidine; 4-hydroxybutyrate^44^**,** while in patients infected with COVID-19 LC–MS analysis showed the presence of bile acids, bilirubin, diacylglycerols, free fatty acid, glucose, glucuronate, glycerol 3-phosphate, kynurenine, lysophosphotidylcholines, malic acid, monosialodihexosylganglioside, phosphatidylcholines, sphingomyelin, triglycerides and tryptophan^43^**.** In urinary tract infections caused by *Escherichia coli* by using ^1^H-NMR and LC–MS, the following metabolites characteristic for this type of infection were selected by examination of urine: acetate, amines, aspartic acid, cadaverine, citrate, glutamic acid, glycine, hippurate, trimethylamine, and trimethylamine n-oxide^45^. In the patients with tuberculosis, the potential biomarkers selected by assessment of serum/plasma samples using LC–MS, FIA-MS, and GC-M methods were amino-acyl tRNA, asparagine, aspartate, citrulline, cysteine, gamma-glutamylglutamine, glutamate, glutamine, histidine, inosine, kynurenine, lysophosphatidylcholines, mannose methionine, sphingolipid, sphingosine-1-phosphate, sulfoxymethionine, tryptophan and urea^45^. In the previous study, we used Fourier-transform infrared spectroscopy (FTIR) to combine hierarchical cluster analysis (HCA) to determine the fragments of infrared spectroscopy (IR) spectra and their corresponding biomolecules of guinea pig sera characteristic for *H. pylori* infection. Specific molecules, which were identified in the composition of the IR spectra of guinea pig sera, included glucose, α2-globulins, IgM, IgG1, transferrin, IgG4, C-reactive protein (CRP), and tumor necrosis factor alfa (TNF-alfa), which facilitated the differentiation between *H. pylori* infected and *H. pylori* uninfected animals^46^**.**

Data obtained in this study using the UPLC-QTOF/MS in conjunction with Fold change analysis and T-test results facilitated selection of 22 metabolites with a signal intensity significantly lower and 48 metabolites with a signal intensity markedly higher in *H. pylori* infected *vs.* *H. pylori* uninfected animals, and 12 metabolites, which delivered significantly higher signals were selected as well differentiating biomarkers associated with *H. pylori* infection. Database searching and literature analysis indicated that these 12 selected metabolites well differentiating animals infected with *H. pylori* from animals uninfected with these bacteria were primarily associated with immune regulation, energy metabolism, lipid/fatty acid metabolism, lipid peroxidation, oxidative stress, and cell signaling, and correlate with the pathogenesis of *H. pylori* infection in humans. The remaining 36 metabolites can potentially be treated as additional markers related to *H. pylori* infection, however their role in the course of *H. pylori* pathogenesis has not been described.

Among selected 12 differentiating metabolites leukotriene B4 is a pro-inflammatory dihydroxy fatty acid derived from arachidonic acid produced by leukocytes involved in the inflammatory response. It promotes the adhesion of leukocytes to the vascular endothelium and activates them to produce pro-inflammatory cytokines and mediators^47^ during chronic inflammation and cancer^48^**.** Hüseyinov et al. showed an elevated level of leukotriene B4 in gastric juice in *H. pylori-*positive children^49^**.**

Lysophospholipid (LPC) exerts biological effects on other cells by stimulating calcium flux, cell proliferation, differentiation, and activation. The LPC-driven migration of glioma cells and mouse fibroblasts but not macrophages was shown in several studies^50–52^**.** Higher levels of LPC induce cyclooxygenase-2 and endothelial nitric oxide synthase (eNOS) expression in endothelial cells, which can have vasoprotective effects via prostacyclin or nitric oxide. LPCs have been shown to elicit several effects on the innate immune system and effectively serve as dual-activity ligand molecules. In particular, LPCs directly activate toll-like receptor (TLR) 4 and TLR-2 without classical TLR ligands. However, LPCs can also inhibit TLR-mediated signaling in the presence of classical TLR ligands, thereby acting as anti-inflammatory molecules. Low levels of LPC during a bacterial or viral infection with TLR-mediated signaling can lead to opposing (inflammatory *vs*. anti-inflammatory) effects and immune dysregulation^31^**.**

Phospholipids (PS) bind to various proteins and activate enzymes, apoptosis, neurotransmission, and synaptic refinement^53^**.** Phosphatidylglycerol (PG) is distributed in the animal cell mitochondria and, when located in lung surfactant, is critical for regulating innate immunity^54,55^**.** PG competes with bacterial lipopolysaccharides to disrupt the signaling pathways dependent on toll-like receptors (TLRs)^56,57^**.** Phospholipids, mainly phosphatidylethanolamine (PE), comprise most *H. pylori* lipids in developing membrane domains^58,59^**.** *H. pylori* also produces outer membrane vesicles (OMVs) that contain various virulence factors that promote the survival of these bacteria in the gastric mucosa^60^**.** *H. pylori* OMVs contain PG, PE, lyso PE (LPE), phosphatidylcholine (PC), lyso PC (LPC), and cardiolipin^60^**.** PS externalization is accompanied by increased permeability of eukaryotic cell membranes^61^**.** *H. pylori* OMVs, due to the induction of gastric barrier instability and dysbiosis, can be translocated to deeper layers of gastric mucosa and in the gut. They may also be translocated to the circulation where, due to interaction with leukocytes, OMVs may affect the activity of these immune cells. Also, direct contact of *H. pylori* with the host cell membranes induces rapid and transient externalization of PS, independently of the apoptotic process at the bacterial attachment site^62^**.** Moreover, *H. pylori* exploits host membrane phosphatidylserine for delivery, localization, and pathophysiological effects of the CagA oncoprotein^62^**.** Therefore, increased levels of lipids, including PG and PS, in the serum samples of guinea pigs infected with *H. pylori* may be potential markers of systemic effects of *H. pylori* infection related to chronic inflammation. Some of the metabolomic biomarkers increased in *H. pylori* infected guinea pigs, such as 20-dihydroxyleukotriene B4 and dihomo-gamma-linolenoylethanolamide is engaged in the peroxidation of lipids, which in such form are involved in the development of vascular disorders. It is worth mentioning that *H. pylori* infection is considered a non-classical risk factor in the development of coronary heart disease^14,15,36,63–65^**.** It has been shown that *H. pylori* LPS increases oxidative stress, which results in lipid peroxidation assessed as an increased level of 4-Hydroxynonenal (4HNE) both in gastric epithelial cells and vascular endothelium^36^**.** Furthermore, *H. pylori* components induced the transformation of macrophages into foam cells in vitro*.* They increased the severity of the metabolic syndrome and hepatic manifestations caused by a high-fat diet in a model of *Cavia porcellus*. The infiltration of inflammatory cells into the vascular endothelium of animals infected with *H. pylori* and exposed to a high-fat diet was observed in conjunction with an increased level of inflammatory markers systemically^39,41^**.**

In this study, the leucine metabolites (Iso leucyl-phenylalanine, N-Lactoylleucine) differentiated the animals infected with *H. pylori* from those uninfected with these bacteria. Felig et al., using the rat model, showed an increased leucine plasma level in these rodents, which was related to insulin resistance^66^ and cardiovascular dysfunction in obese rats^67^**.** Interestingly, increased concentration of plasma leucine was associated with impaired endothelium-dependent relaxation in Zucker diabetic fatty rats^68^ and with reduced energy usage in rats with diet-induced obesity^69,70^**.** The effect of affected endothelium relaxation was also shown in humans with hyperglycemia^71^. Our previous study showed diminished vascular relaxation in guinea pigs receiving a high-fat diet infected with *H. pylori*^39,41^**.**

In the present study, deoxycholic acid (DCA) differentiated guinea pigs infected with *H. pylori* from those not infected. Frazier et al. showed that elevated serum level of DCA, a secondary bile acid, was associated with vascular calcification in patients with chronic kidney disease (CKD)^72^**.** DCA causes numerous harmful effects, including decreased insulin sensitivity and immune dysregulation^73^ and potential cardiovascular toxicity by promoting vascular calcification^73^**.** Therefore, increased levels of leucine and/or DCA in the serum may be potential markers of vascular endothelium dysfunction.

5-Methoxytryptophol is an indoleamine synthesized from the pineal gland hormone serotonin and, together with melatonin, is involved in regulating daily rhythms. 5-Methoxytryptophol is highest during the day, while melatonin is highest at night. 5-methoxytryptophol-mediated receptors play a role in regulating cerebral artery contractility, intra arrhythmia, and the regulation of renal function. 5-Methoxytryptophol also shows immunomodulatory, antioxidant, and anxiolytic properties with different mechanisms. Environmental factors other than light, including insulin-induced hypoglycemia, can override the inhibitory effects of light and accelerate melatonin synthesis^45,46^**.** In this study, 5-Methoxytryptophol was a differentiating metabolite in the sera of H. pylori-infected guinea pigs. A decrease in the serum levels of melatonin synthesizing enzymes is seen in patients with symptomatic *H. pylori* infection, and potentially, the processes regulated by 5-methoxytryptophol/melatonin might be affected in these patients. Luo et al., showed that melatonin used to immunize mice infected with *H. pylori* could eradicate these bacteria. However, the exact molecular mechanism is unknown. It can be that melatonin treatment of mice infected with *H. pylori* resulted in a decreased count of regulatory lymphocytes (CD4 + CD25 + Foxp3 + Treg) in the spleen and diminished secretion of transforming growth factor-beta (TGF-beta), regulatory cytokine, on TLR-4 and TLR-2 dependent manner^74^**.** It has been suggested that different neurological disorders, such as Parkinson’s disease, Alzheimer’s disease, Guillain-Barré syndrome, and multiple sclerosis, are linked to *H. pylori* infection. In some of them, sleep disruption, nightly restlessness, sundowning, and other circadian disturbances are frequently seen^75^**.** In animal models of Alzheimer’s disease, *H. pylori* infection localized in the stomach induced neuroinflammation, and the potential role of outer membrane vesicles has been suggested in developing this phenomenon^76^**.**

GABA is the principal inhibitory neurotransmitter in the central nervous system. Both the downregulation of GABA production and excessive release of this neurotransmitter can drive neurological disorders^77^**.** However, little is known about the potential link between *H. pylori* infections and GABA modulation. It has been shown that *H. pylori* infection releases several neurotransmitters, such as acetylcholine, adrenaline, noradrenaline, serotonin, and dopamine. Moreover, *H. pylori* infection might lead to axonal/neuronal damage, production of free radicals, and changes in neuropeptide expression, such as vasoactive intestinal peptides^78^. In this study, elevated levels of GABA were observed in serum samples of *H. pylori*-infected guinea pigs. It has been reported that GABA has antioxidant and anti-inflammatory action by regulating major inflammatory events and immune cell activities^79^**.** Considering those GABA activities, excessive concentration of GABA in our model of *H. pylori* infection in guinea pigs may be a part of the host response against exposure to *H. pylori*.

This study shows that the above selected serum metabolites potentially may be valuable biomarkers for predicting and monitoring the systemic consequences of *H. pylori* infection.

## Conclusions

Using metabolomic analysis of serum samples alongside ROC statistical analysis, we selected 12 signatures corresponding to *H. pylori* infection which facilitated differentiation of serum samples of *H. pylori* infected animals from the sera of control animals, which were not infected with these bacteria. The selected metabolites included: 20-dihydroxyleukotriene B4 (HMDB0012635); PGD2 ethanolamide (HMDB0013629); L.L-cyclo(leucylprolyl) (HMDB0034276); LysoPC(P-16:0/0:0) (HMDB0010407); PS(18:2(9Z.12Z)/18:2(9Z.12Z)) (HMDB0012402); PG(18:2(9Z.12Z)/22:4(7Z.10Z.13Z.16Z)) (HMDB0010656); N-Lactoylleucine (HMDB0062176); Isoleucyl-Phenylalanine (HMDB0028914); 5-Methoxytryptophol (HMDB0001896); Deoxycholic acid 3-glucuronide (HMDB0002596); PC(16:1(9Z)/14:1(9Z)) (HMDB0007999); Gamma-Aminobutyric Acid (HMDB0000112). Further investigation involving a higher number of serum samples will allow for the standardization of methodology used in this study for analysis of guinea pig sera regarding *H. pylori* infection, which is accompanied by the development of chronic inflammatory responses, which potentially may drive deleterious systemic effects. Knowledge from the guinea pig model will help understanding the processes occurring during *H. pylori* infection in humans and potentially will facilitate the selection of metabolic biomarkers, which will improve the diagnostic and monitoring of systemic effects related to this infectionn.

## Supplementary Information

Supplementary Information.

## Data Availability

All data generated or analyzed during this study are included in this published article or supplementary file. The datasets used and/or analyzed during the current study are available from the corresponding author on reasonable request.
