# Supplementary material for: Untargeted metabolomic profiling for identifying systemic signatures of helicobacter pylori infection in a guinea pig model
Source: Sci Rep. 2025 Apr 15;15:12889. doi: 10.1038/s41598-025-98016-w (PMC12000522; doi:10.1038/s41598-025-98016-w)
Supplement: Supplementary file 1 — Supplementary Information. [file 41598_2025_98016_MOESM1_ESM.docx]

Supplementary Information

**Untargeted metabolomic profiling as a new tool to identify systemic signatures of *Helicobacter pylori* infection in a guinea pig model**

Weronika Gonciarz, Lucyna Kozłowska, Joanna Róg, Magdalena Chmiela

**Table S1.** The optimized gradient elution procedure (positive mode)

| **Time** | **Flow (mL/min)** | **%A1** | **%B1** | **Curve** |
| --- | --- | --- | --- | --- |
| Initial | 0.3 | 99 | 1 | Initial |
| 1.00 | 0.3 | 85 | 15 | 6 |
| 6.00 | 0.3 | 20 | 80 | 6 |
| 10.00 | 0.3 | 0 | 100 | 6 |
| 11.00 | 0.3 | 0 | 100 | 6 |
| 12.00 | 0.3 | 99 | 1 | 6 |
| 15.00 | 0.3 | 99 | 1 | 6 |

**Table S2.** The optimized gradient elution procedure (negative mode)

| **Time** | **Flow (mL/min)** | **%A1** | **%B1** | **Curve** |
| --- | --- | --- | --- | --- |
| Initial | 0.25 | 99 | 1 | Initial |
| 1.00 | 0.25 | 75 | 25 | 6 |
| 2.00 | 0.25 | 55 | 45 | 6 |
| 3.00 | 0.25 | 40 | 60 | 6 |
| 4.00 | 0.25 | 26 | 74 | 6 |
| 10.00 | 0.25 | 20 | 80 | 6 |
| 11.00 | 0.25 | 0 | 100 | 6 |
| 12.00 | 0.25 | 99 | 1 | 6 |
| 15.00 | 0.25 | 99 | 1 | 6 |


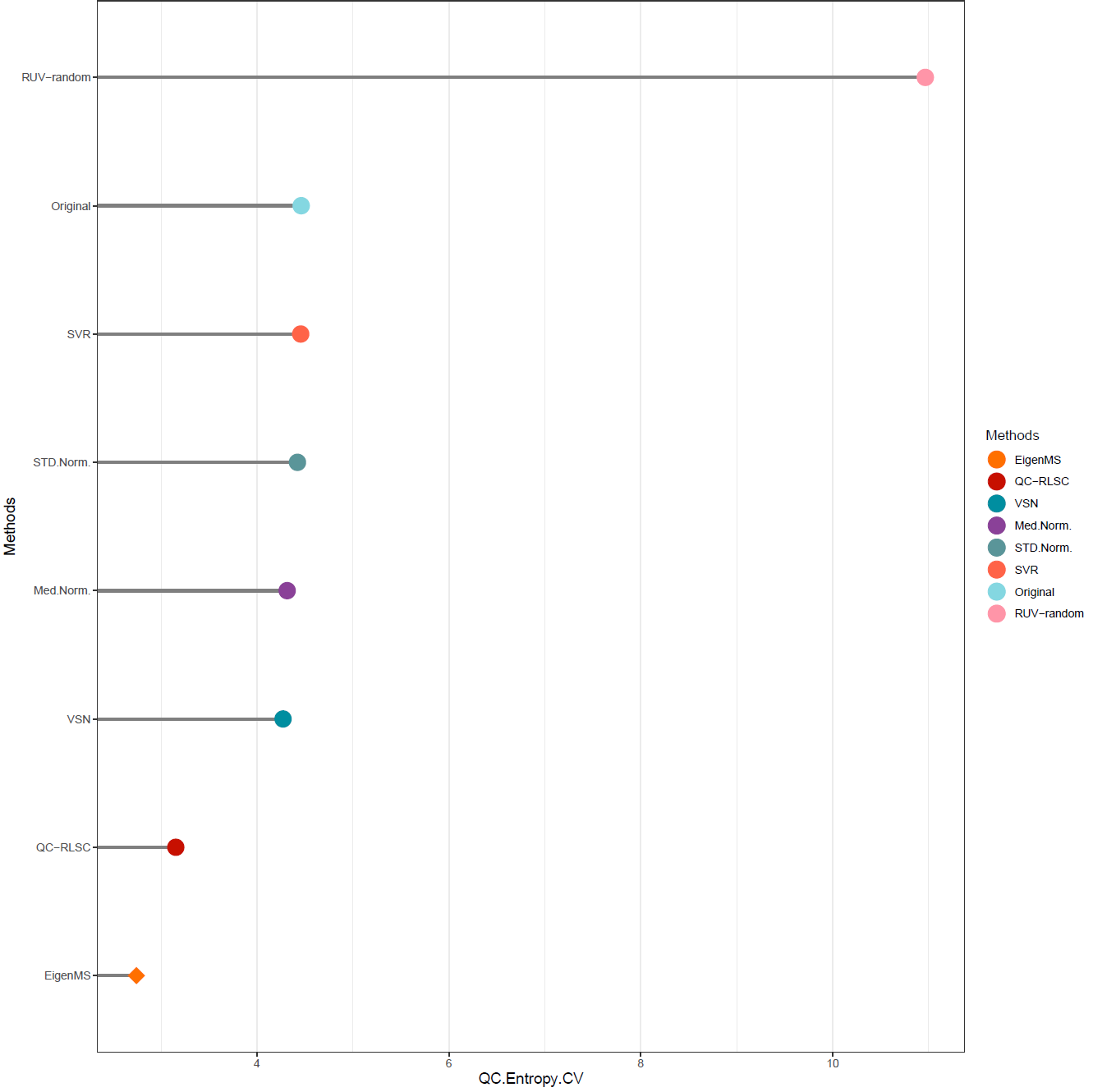


**Figure S1.** Summary results of coefficients of variation (CV) of entropy in QC samples with respect to no normalization and seven normalization methods in negative MS mode.

Abbreviations: EigenMS - singular value decomposition-based normalization; QC-RLSC - QC sample-based support vector regression; VSN - variance-stabilizing normalization; MedNorm - median normalization; STD.Norm. - standard normalization; Original – no normalization; RUV-random - removal of unwanted variation-random normalization


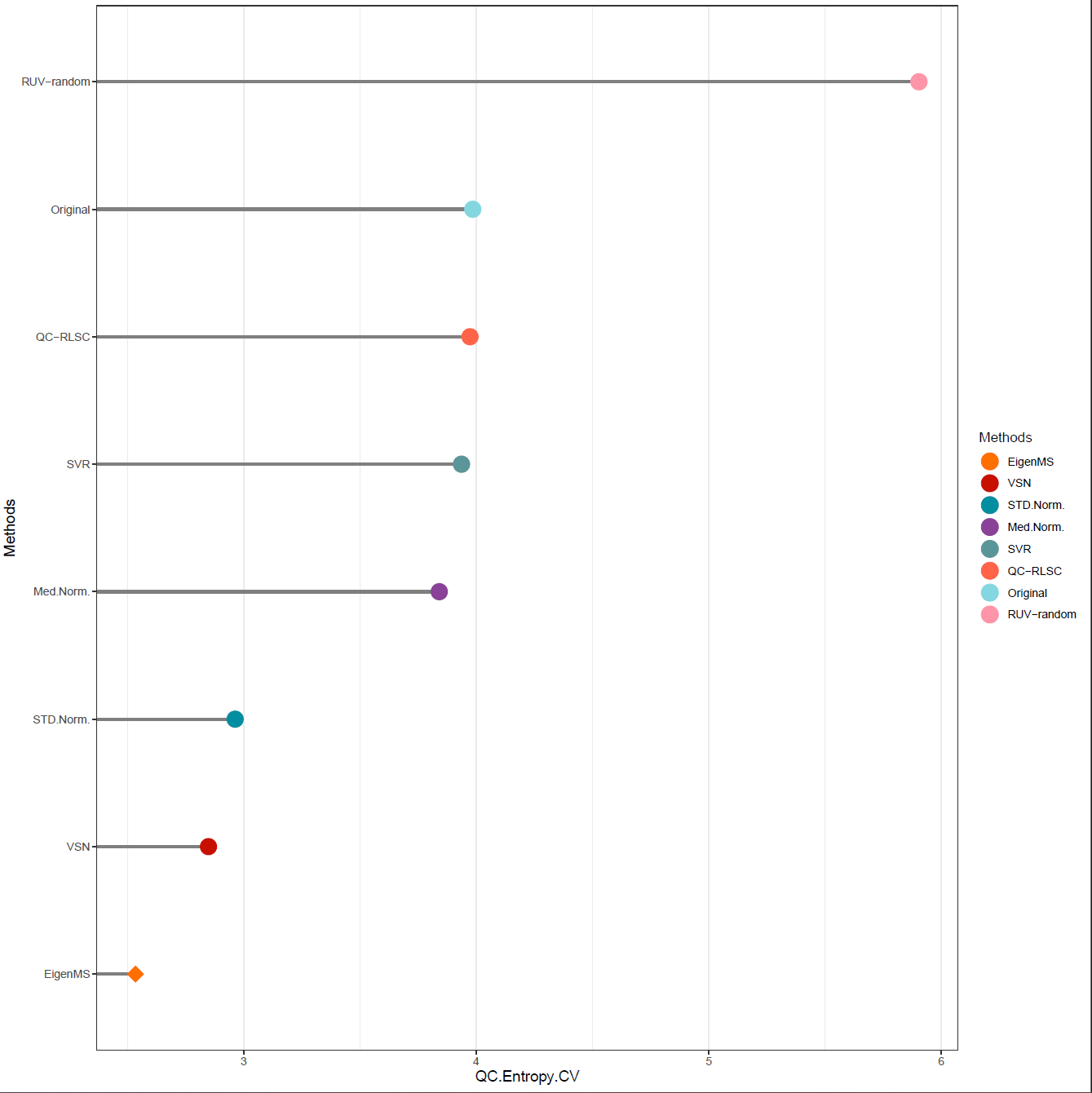


**Figure S2.** Summary results of coefficients of variation (CV) of entropy in QC samples with respect to no normalization and seven normalization methods in positive MS mode.

Abbreviations: EigenMS - singular value decomposition-based normalization; QC-RLSC - QC sample-based support vector regression normalization; VSN - variance-stabilizing normalization; MedNorm - median normalization; STD. Norm. - standard normalization; Original – no normalization; RUV-random - removal of unwanted variation-random normalization

**Table S3** AUC, Log2 FC and T-tests and K-Means Clusters for univariate biomarker analysis.

| **No.** | **Name** | **AUC** | **T-tests** | **Log2 FC** | **Cluster** |
| --- | --- | --- | --- | --- | --- |
| 1 | 20-dihydroxyleukotriene B4 (HMDB0012635) | 0.97705 | 4.0903E-12 | 0.65845 | 1 |
| 2 | PGD2 ethanolamide  (HMDB0013629) | 0.98926 | 2.523E-15 | 0.72803 | 1 |
| 3 | L.L-Cyclo(leucylprolyl)  (HMDB0034276) | 1.0 | 6.0626E-34 | 0.75171 | 4 |
| 4 | LysoPC(P-16:0/0:0)  (HMDB0010407) | 1.0 | 2.1244E-20 | 0.70008 | 4 |
| 5 | PS(18:2(9Z.12Z)/18:2(9Z.12Z)) (HMDB0012402) | 0.99512 | 3.4537E-16 | 0.69599 | 1 |
| 6 | PG(18:2(9Z.12Z)/22:4(7Z.10Z.13Z.16Z)) (HMDB0010656) | 1.0 | 4.4623E-24 | 0.67825 | 1 |
| 7 | N-Lactoylleucine  (HMDB0062176) | 0.99512 | 3.4223000000000004E-19 | 0.67573 | 1 |
| 8 | Isoleucyl-Phenylalanine  (HMDB0028914) | 0.99316 | 7.6823E-20 | 0.66953 | 5 |
| 9 | 5-Methoxytryptophol  (HMDB0001896) | 1.0 | 9.9341E-46 | 0.63712 | 4 |
| 10 | Deoxycholic acid 3-glucuronide (HMDB0002596) | 1.0 | 9.9449E-30 | 0.63634 | 4 |
| 11 | PC(16:1(9Z)/14:1(9Z))  (HMDB0007999) | 0.99805 | 2.0959E-23 | 0.58861 | 1 |
| 12 | gamma-Aminobutyric acid (HMDB0000112) | 0.94238 | 1.3279E-11 | 0.60811 | 1 |


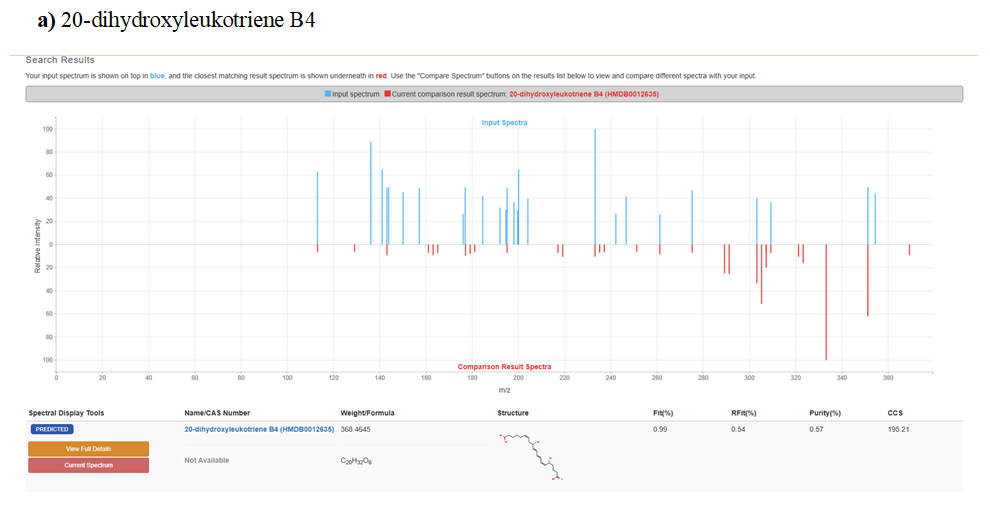


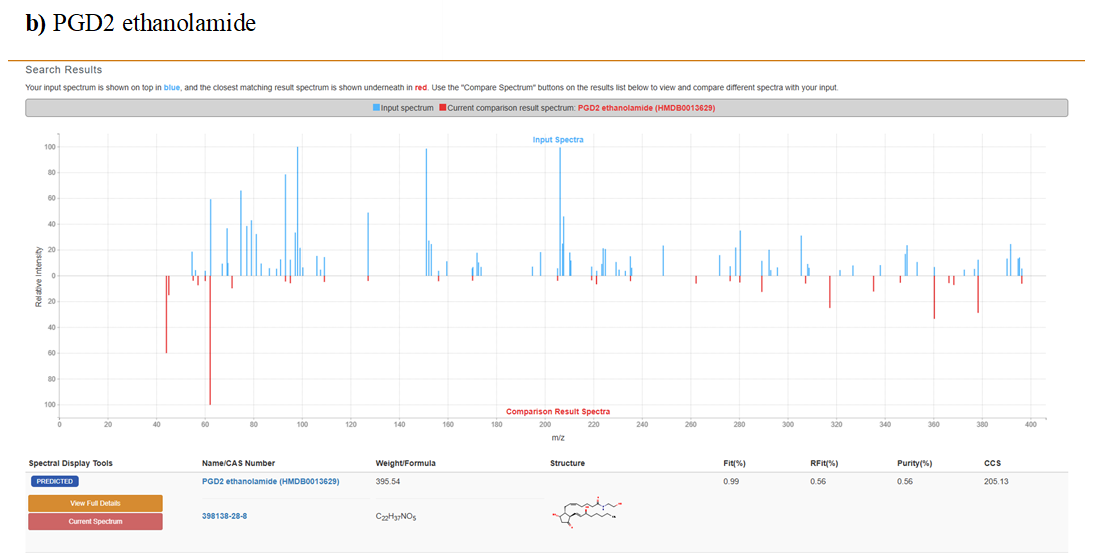


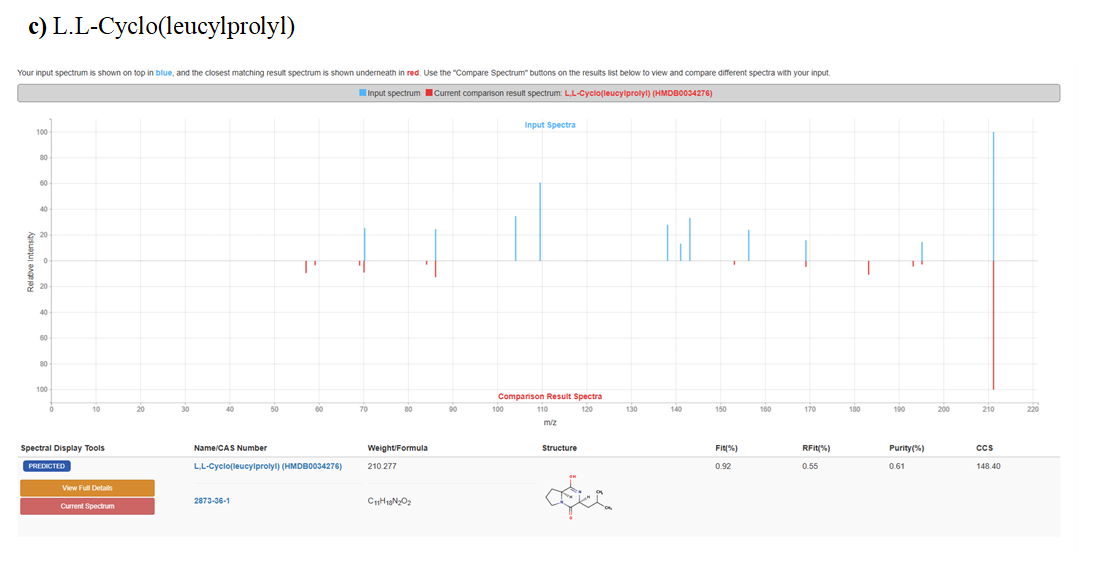


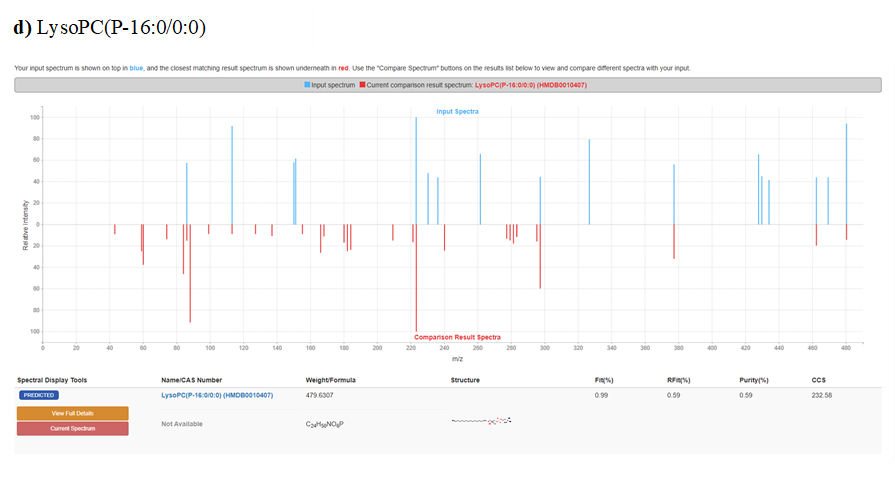


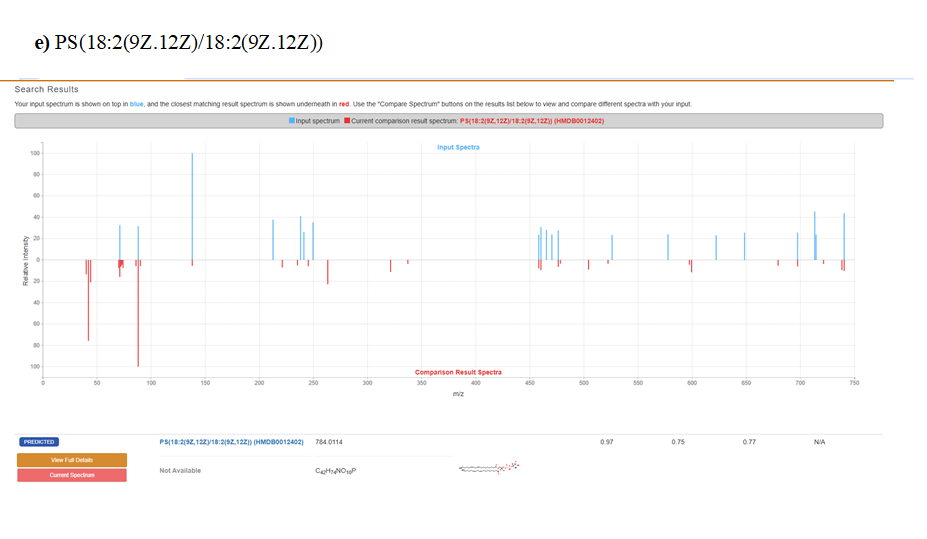


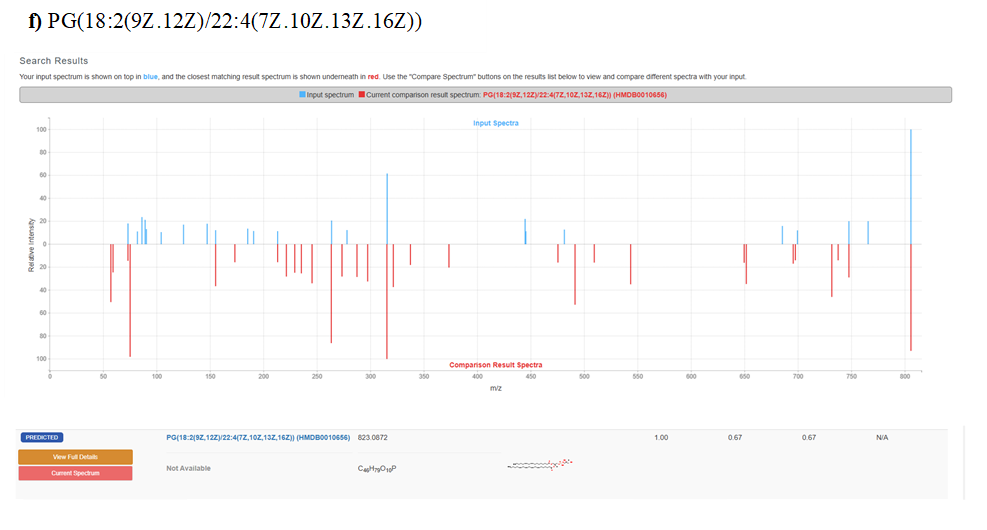


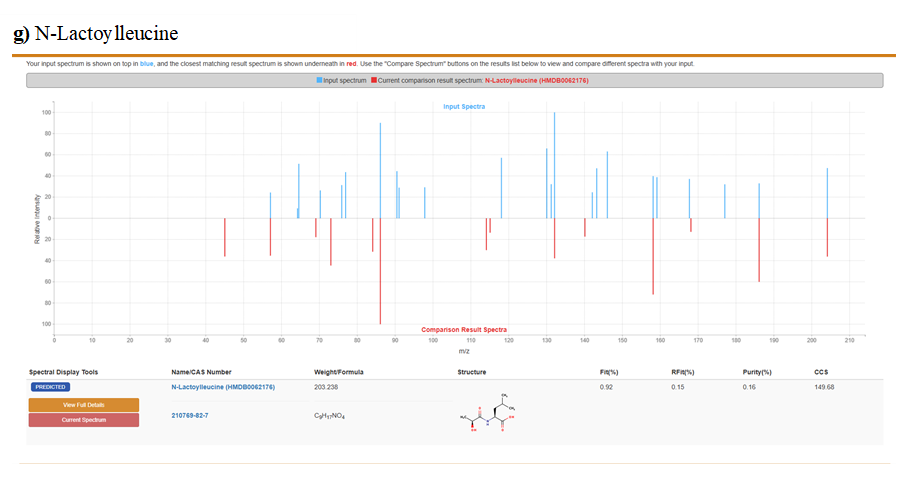


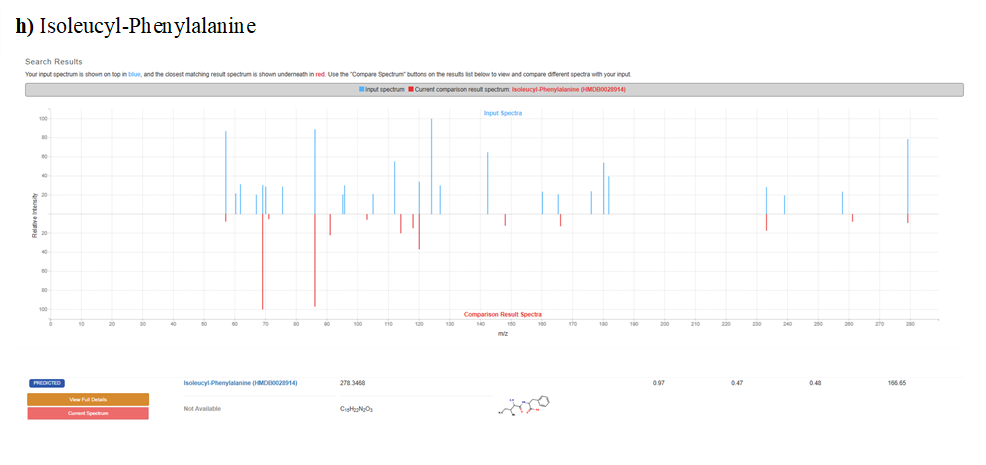


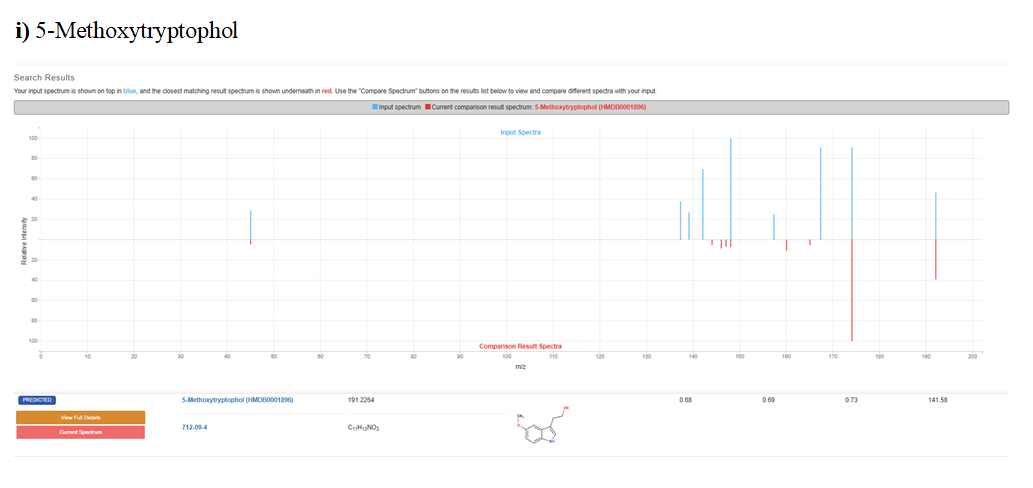


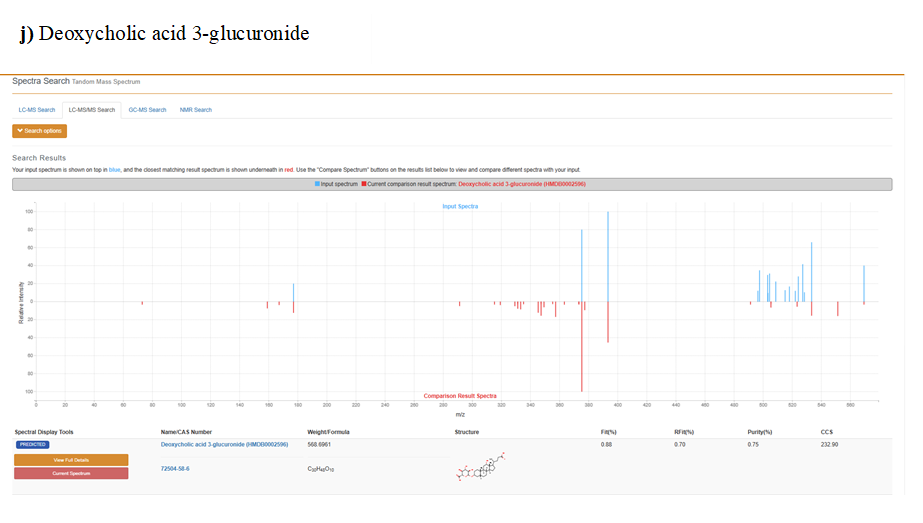


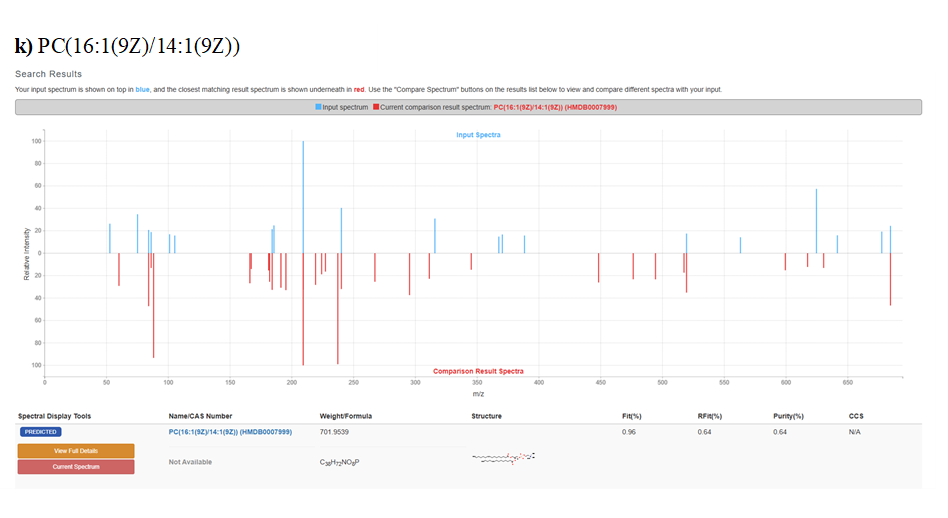


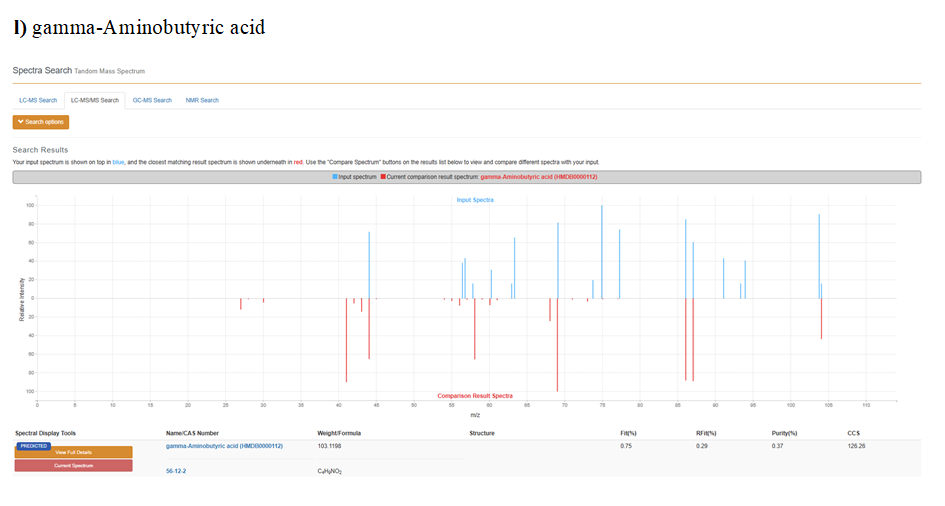


**Figure S3** MS/MS spectra of putatively annotated metabolites - our input spectra are shown on top in blue, and the closest matching result spectra are shown underneath in red.
